# Does Pathologic Response Equate to Clinical Response Following SABR for Early-Stage NSCLC?

**DOI:** 10.3389/fonc.2019.00551

**Published:** 2019-06-21

**Authors:** Eric D. Brooks, Vivek Verma, Joe Y. Chang

**Affiliations:** ^1^Department of Radiation Oncology, University of Texas M.D. Anderson Cancer Center, Houston, TX, United States; ^2^Department of Radiation Oncology, Allegheny General Hospital, Pittsburgh, PA, United States

**Keywords:** missile, lung cancer, NSCLC, SBRT, pathologic complete response, PCR, surgery, lobectomy

We applaud the recent efforts of Palma et al. for prospectively addressing a novel issue regarding pathologic response following stereotactic ablative radiotherapy (SABR) for early-stage non-small cell lung cancer (1). This was a phase II trial evaluating the addition of neoadjuvant SABR prior to resection for purposes of evaluating the pathologic complete response (pCR) rate following SABR. This is of academic interest because the vast majority of SABR candidates are inoperable, and hence the pathologic response of SABR is largely not able to be assessed. The primary findings were a low (60%) pCR, highly discordant with the well-validated ≥90% local control (LC) in the irradiated lesion following modern, volumetric image-guided SABR (2–4). Although the low pCR may be concerning for many readers, there are several assuaging caveats worth highlighting.

First, the treatment paradigms of this study may allow for further optimization. For instance, SABR dosing in 25% of patients (e.g., 60 Gy/8 fractions) was of relatively lower biologically effective dose (BED) than employed at other institutions. This is noteworthy because BED can be further escalated by means of several treatment planning nuances, which is particularly important for lesions larger than 3 cm (5).

More importantly, the value and accuracy of histopathology at 10 weeks following SABR is evidently quite poor, and thus should not reflect the consistently high clinical LC with long-term follow up after SABR. This discrepancy is analogous to the situation regarding post-SABR positron emission tomography (PET), which may be unreliable even 1 year from SABR (6). Thoracic radiation oncologists are undoubtedly aware of scenarios (albeit uncommon) in which results of a 3–6-month post-SABR PET lead to a positive biopsy, but no treatment is delivered owing to comorbidities, and yet long-term follow-up reveals no recurrences (Figure 1). This may be explained by basic radiobiological principles; tumor cells can appear viable on histopathology but in fact are dead, dying, or senescent from lethal chromosomal damage. This is reflected by higher pCR rates with increasing time from hypofractionated/fractionated radiotherapy to resection of rectal and esophageal cancers (7–9). Moreover, the trial implies that LC is achieved not only by direct (SABR-mediated) tumoricide, but also lends credence to immune-based destruction as a vital component thereof. Because the latter can continue to occur past 10 weeks (the time of resection), the trial unfortunately eliminates this concept from the equation. Nevertheless, this issue will be better addressed (along with the preponderance of post-SABR out-of-field failures) by means of ongoing trials delivering concurrent and adjuvant immunotherapy with SABR (I-SABR, NCT03110978; NCT03446547; NCT03924869; NCT03833154). Although the observed pCR rate is perhaps anxiety-provoking, readers are recommended to continue to rely on the well-corroborated clinically-measured LC rates. Those findings should also not be a deterrent to enrollment onto important SABR trials such as VALOR, STABLE-MATES, SABRTooth, and POSITILV. Although academically interesting, performing two local therapies for an early-stage neoplasm for which either modality displays impressively high LC remains questionable. The rate of positive margins is exceedingly low in sublobar approaches (10) (and even lower with lobectomies) that neoadjuvant SABR would not provide a meaningful benefit thereof. Additionally, for reasons mentioned above, if the theoretical “selling point” of adding neoadjuvant SABR relates to promoting abscopal responses, then perhaps surgery should not be performed in the first place (11).

**Figure 1:**
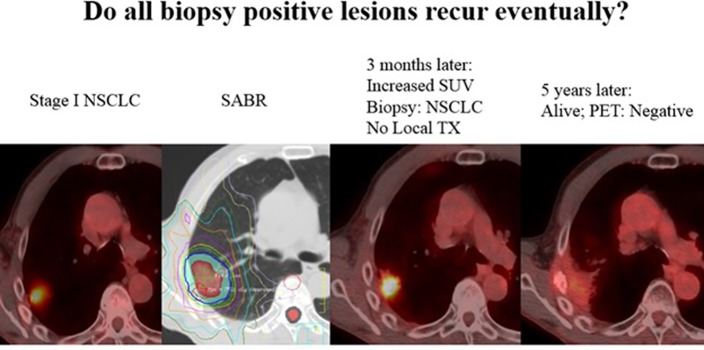
Not all “positive” biopsies months after SABR lead to recurrence. Here, a 71 year old male with stage I NSCLC was treated with SABR, 50Gy in 4 fractions. A routine surveillance PET/CT three months later after SABR showed increased SUV uptake. A biopsy was performed and was positive for “residual cancer.” The patient was sent for evaluation of surgery and thermal ablation but neither were recommended due to poor performance status. The patient was followed, and 5 years later his tumor has disappeared and he has been without disease or any signs of local recurrence. PET uptake has also resolved without any treatment. Although a biopsy and PET may suggest viable disease, tumor cells can often be dead/dying after SABR and lead to no recurrence.

## Author Contributions

All authors listed have made a substantial, direct and intellectual contribution to the work, and approved it for publication.

### Conflict of Interest Statement

The authors declare that the research was conducted in the absence of any commercial or financial relationships that could be construed as a potential conflict of interest.
